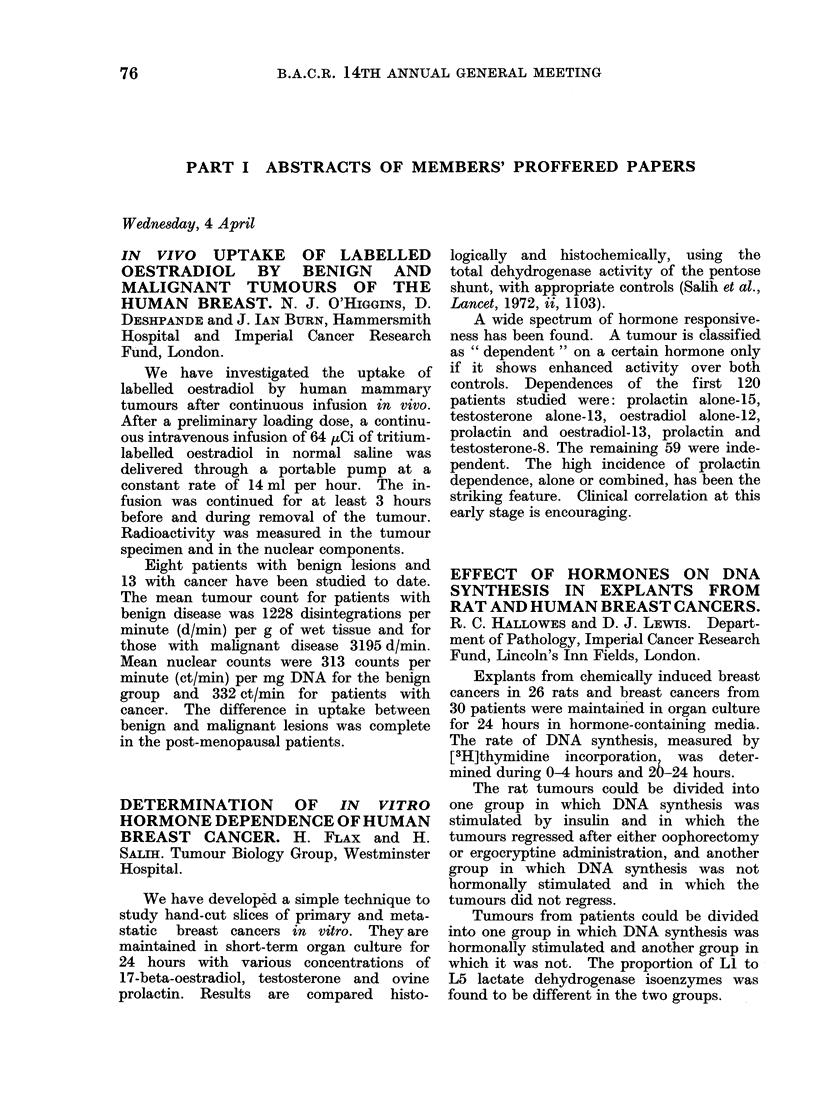# In vivo uptake of labelled oestradiol by benign and malignant tumours of the human breast.

**DOI:** 10.1038/bjc.1973.73

**Published:** 1973-07

**Authors:** N. J. O'Higgins, D. Deshpande, J. I. Burn


### PART I ABSTRACTS OF MEMBERS' PROFFERED PAPERS

*Wednesday, 4 April*

### *IN VIVO* UPTAKE OF LABELLED OESTRADIOL BY BENIGN AND MALIGNANT TUMOURS OF THE HUMAN BREAST.

N. J. O'Higgins, D. Deshpande and J. Ian Burn, Hammersmith Hospital and Imperial Cancer Research Fund, London.

We have investigated the uptake of labelled oestradiol by human mammary tumours after continuous infusion *in vivo*. After a preliminary loading dose, a continuous intravenous infusion of 64 $\mu$Ci of tritium-labelled oestradiol in normal saline was delivered through a portable pump at a constant rate of 14 ml per hour. The infusion was continued for at least 3 hours before and during removal of the tumour. Radioactivity was measured in the tumour specimen and in the nuclear components.

Eight patients with benign lesions and 13 with cancer have been studied to date. The mean tumour count for patients with benign disease was 1228 disintegrations per minute (d/min) per g of wet tissue and for those with malignant disease 3195 d/min. Mean nuclear counts were 313 counts per minute (ct/min) per mg DNA for the benign group and 332 ct/min for patients with cancer. The difference in uptake between benign and malignant lesions was complete in the post-menopausal patients.